# The Ankle‐GO™ score predicts return to sport after surgically treated unstable syndesmotic injuries

**DOI:** 10.1002/jeo2.70907

**Published:** 2026-09-14

**Authors:** Andrei Corbu, Ronny Lopes, Eugénie Valentin, Mette Renate Andersen, François Fourchet, Gauthier Rauline, Brice Picot, Alexandre Hardy

**Affiliations:** ^1^ Clinique du Sport Paris France; ^2^ Centre Orthopédique Santy, FIFA Medical Center of Excellence Hôpital Privé Jean Mermoz, Groupe Ramsay Lyon France; ^3^ Department of Orthopedic Surgery Aleris Hospital Tromsø Tromsø Norway; ^4^ Physiotherapy Department La Tour Hospital Swiss Olympic Medical Centre Meyrin Switzerland; ^5^ French Society of Sports Physical Therapy (SFMKS Lab) Pierrefitte‐sur‐Seine France; ^6^ Inter‐University Laboratory of Human Movement Biology LIBM, EA 7424 Savoie Mont‐Blanc University Le Bourget‐du‐Lac France

**Keywords:** Ankle‐GO, functional tests, return‐to‐sport, syndesmotic injuries, stabilization

## Abstract

**Purpose:**

Objective return to sport (RTS) criteria following syndesmotic stabilization are limited. The purpose of this study was to explore the discriminative and predictive ability of the Ankle‐GO™ score for RTS status following syndesmotic stabilization.

**Methods:**

At 3 and 6 months postoperatively, 50 patients were assessed using the Ankle‐GO^TM^ score, which consists of four functional tests and two self‐reported questionnaires. At 6 months, patients reported their RTS status using three possible responses: no RTS, RTS at a lower level or RTS at the same or a higher level than before injury. Responsiveness of Ankle‐GO^TM^ was calculated by comparing 3‐ and 6‐month scores using the Wilcoxon signed‐rank test for paired samples. Discriminant validity was calculated by comparing 6‐month scores between patients according to their RTS status. Area under the receiver operating curve (AUC) and logistic regression with odds ratios (ORs) and 95% confidence intervals (CIs) were used to determine the ability of the 3‐month Ankle‐GO^TM^ score to predict RTS status at 6 months.

**Results:**

At 6 months, 80% of patients (40) RTS, with 58% of these (29 patients) at their pre‐injury level. The median Ankle‐GO™ score increased significantly from 13 points (interquartile range [IQR], 10.0–16.0) at 3 months to 19 points (IQR, 16.0–22.0) at 6 months (*p* < 0.0001). The 3‐month score showed excellent predictive ability of RTS status at 6 months (AUC = 0.93; 95% CI: 0.86–1.00). Patients scoring above 12 points are more likely to RTS (OR = 42.78, 95% CI: 2.33–785.93, *p* < 0.01). A score above 14 points predicted RTS to the preinjury level (AUC = 0.83, 95% CI: 0.73–0.94; OR = 9.44, 95% CI: 2.66–40.7, *p* < 0.01).

**Conclusion:**

The Ankle‐GO™ demonstrated promising discriminative and predictive ability for assessing RTS status after syndesmotic stabilization. Further studies are needed to confirm these results and the ability of this tool to identify patients who will suffer recurrence.

**Level of Evidence:**

Level II, prospective cohort study.

## Graphical abstract

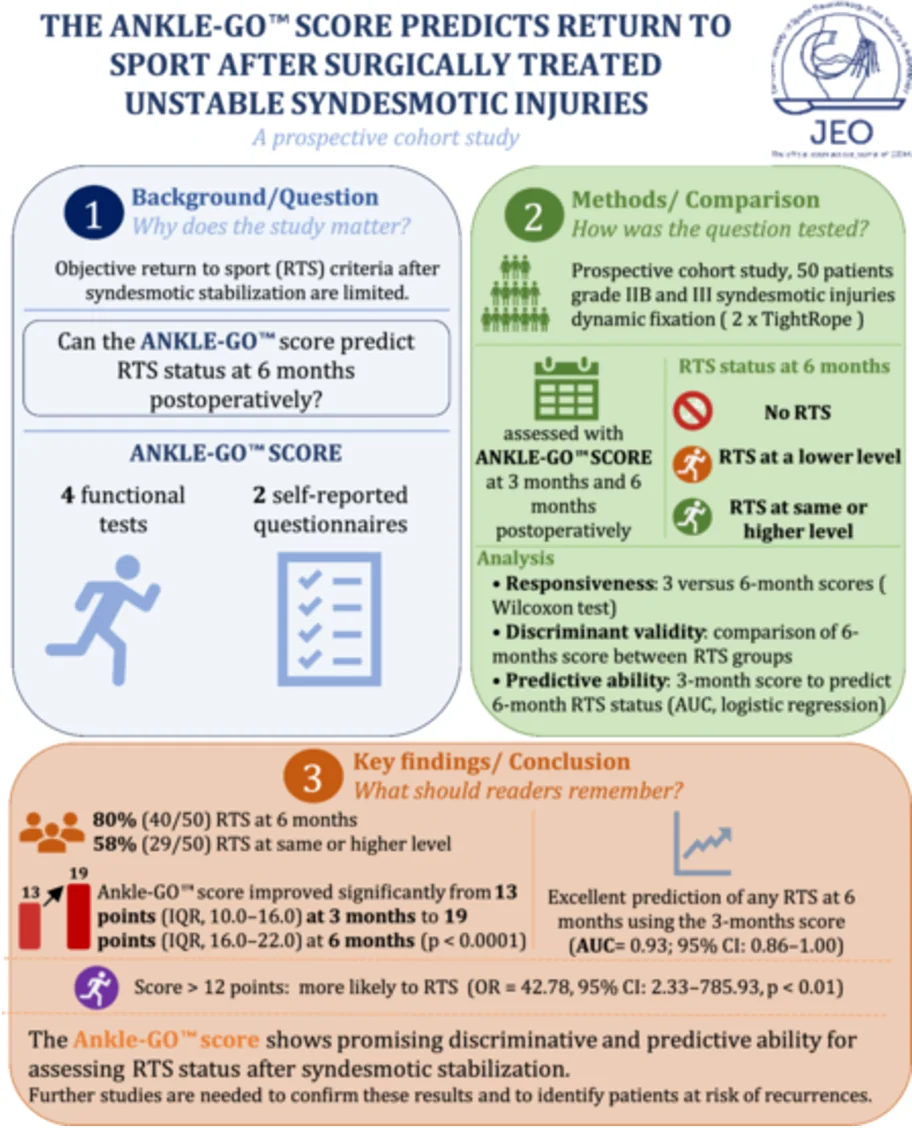

**Abbreviations**
ADL: activity of daily living
AITFL: anterior inferior tibiofibular ligament
ALR‐RSI: Ankle Ligament Reconstruction–Return to Sport after Injury
AOFAS: American Orthopedic Foot & Ankle Society
AP: anteroposterior
AUC: area under the curve
BMI: body mass index
CI: confidence interval
COSMIN: COnsensus‐based Standards for the selection of health Measurement INstruments
ESSKA‐AFAS: European Society of Sports Traumatology
  Knee Surgery and Arthroscopy–Ankle and Foot Associates
F8T: figure‐of‐8 hop test
FAAM: Foot and Ankle Ability Measure
IOL: interosseous ligament
IQR: interquartile range
MRI: magnetic resonance imaging
mSEBT: modified version of the Star Excursion Balance test in percentages
NPV: negative predictive value
OR: odds ratio
PAASS: Pain
  Ankle impairments, Athlete perception, Sensorimotor control and Sport/functional performance
PITFL: posterior inferior tibiofibular ligament
PPV: positive predictive value
PROMs: patient‐reported outcome measures
PROs: patient‐reported outcomes
ROC: receiver operating characteristic curve
ROM: range of motion
RTS: return to sport
SD: standard deviation
SHT: side hop test
SLS: single‐leg stance test

## INTRODUCTION

Acute ankle sprains are among the most frequent musculoskeletal injuries and can be a cause of persistent disability [19, 21]. It is estimated that their incidence is even higher in the active sporting population, accounting for 16%–40% of all the sports injuries [26, 27]. Syndesmotic lesions occur in 1%–18% of ankle sprains, with a higher incidence in the athletic population [24, 36, 59]. Whether associated with fractures or occurring as purely ligamentous lesions, these injuries may cause persistent disability, require longer rehabilitation periods and delay return to sport (RTS) compared with lateral ankle sprains [21, 25, 36, 56, 62]. The European Society of Sports Traumatology Knee Surgery and Arthroscopy–Ankle and Foot Associates (ESSKA‐AFAS) consensus emphasized the importance of differentiating stable from unstable acute syndesmotic lesions because unstable syndesmotic injuries require operative treatment [57]. Although there is no uniform classification system for syndesmotic injuries, one of the most frequently employed is represented by the West Point Ankle Grading System [8, 21, 33, 54, 57]. Grade IIB injuries, which are dynamically unstable, and Grade III injuries require surgical stabilization [8, 33].

RTS in patients with conservatively treated acute Grade I injuries is typically expected to occur between 2 and 8 weeks but may take 9–20 weeks or longer in patients with unstable Grade II or III lesions [13, 26, 36, 37, 41, 49, 62]. Although individual studies have explored potential predictors of RTS, no validated predictive model or comprehensive scoring system has yet been established for patients undergoing syndesmotic surgery [13, 28, 37, 41, 49, 56, 62]. In addition to clinical criteria (pain, tenderness, swelling), recent literature suggests that recovery of strength, neuromuscular control, functional and sport‐specific performance as well as psychological readiness should also be integrated into RTS decision‐making [2, 4, 10, 25, 51, 52, 61].

The Ankle‐GO™ score was initially developed and validated to discriminate and predict RTS outcomes after lateral ankle sprains, but subsequent studies have shown its validity following Achilles tendon repair and lateral ankle reconstruction for chronic ankle instability [23, 32, 46]. In addition, the objective functional tests involve axial loading of the ankle in different positions, which may place stress on the syndesmotic complex in multiple directions, similar to that encountered in ligamentous syndesmotic injuries.

The purpose of this study was therefore to evaluate the ability of the Ankle‐GO^TM^ score to discriminate and predict RTS status in patients surgically treated for unstable acute syndesmotic injuries. The hypothesis was that the Ankle‐GO™ score would demonstrate good discriminative and predictive ability for RTS after dynamic fixation of unstable ligamentous syndesmotic injuries.

## MATERIALS AND METHODS

### Study design

This prospective multicenter observational cohort study enroled consecutive eligible patients with acute unstable ligamentous syndesmotic injuries who presented at either Sports Clinic Paris or Orthopedics Centre Santy Lyon between November 2024 and May 2025 and underwent ankle arthroscopy and dynamic syndesmosis stabilization. The study was approved by the Conseil d'Orientation Scientifique Ramsay Santé, Comité d'Ethique (IRB ID: IRB00010835; approval reference: COS‐RGDS‐2025‐07‐005‐HARDY‐A) and was conducted in accordance with the ethical principles of the 1964 Declaration of Helsinki. All participants provided informed consent at the initial presentation. Surgery was performed by two experienced surgeons (A. H. and R. L.) in accordance with the principles of the dynamic stabilization technique [3]. Patients completed the Ankle‐GO^TM^ score assessment at 3 and 6 months postoperatively. Their performance was independently rated by two trained therapists who were blinded to each other's assessments.

Only active adult patients who participated in recreational, amateur or professional sports and wished to RTS at their pre‐injury level of play were included in the study. The surgery had to be scheduled in the acute period within 6 weeks after a first episode of an unstable ligamentous syndesmotic injury as defined by: documented anterior inferior tibiofibular ligament (AITFL) lesion on magnetic resonance imaging (MRI) with a positive external rotation test and an association of any of the following signs: positive squeeze test, deltoid ligament injury on MRI, suspicion of widened syndesmosis and tenderness along the anterior interosseus membrane extending more than 6 cm proximal to the ankle joint [8, 36]. Exclusion criteria were (i) the presence of osteochondral lesions or ankle joint fractures; (ii) a history of previous syndesmotic injury and (iii) patients with non‐acute ligamentous syndesmotic injuries (that had occurred more than 6 weeks earlier).

### Surgical technique and postoperative rehabilitation protocol

Surgical interventions were performed under spinal or general anaesthesia and started with an arthroscopic examination of the ankle through the standard anteromedial and anterolateral arthroscopic portals. Both surgeons used the same techniques, detailed below, for syndesmotic stabilization and arthroscopic deltoid ligament repair. Osteochondral lesions and injuries to the deltoid ligament, AITFL and the interosseous ligament (IOL) were documented. When an associated deltoid ligament lesion was identified, debridement was performed and the anterior fascicles of the ligament were reinserted using a 2.5‐mm DX FiberTack Knotless Anchor (Arthrex).

Syndesmotic stabilization was performed through a mini‐lateral approach of the fibula. Instability was confirmed with an external rotation stress test under fluoroscopy and by intraoperative passage of a 4.0‐mm shaver through the distal syndesmosis. Reduction of the syndesmosis was performed and maintained subsequently. Following periosteal elevation, two slightly divergent Knotless Syndesmosis TightRope Implant Systems (Arthrex) were inserted after predrilling four cortices in a 20° to 30° lateral to anteromedial direction relative to the coronal plane under fluoroscopic guidance. The first implant was positioned approximately 1.5 cm proximal to the ankle joint, and the second implant was positioned 2 cm proximal to the first implant. Their position was determined under fluoroscopy before cortical drilling. Once the Tightropes were properly positioned on the tibia, the systems were tensioned and a knot was tied over each button on the fibula while maintaining the foot in a neutral position. Compared with screw fixation, dynamic suture‐button stabilization has been associated with more physiological motion, favourable functional outcomes and fewer complications [14]. Although there are concerns about potential lack of stability with single‐button fixation, particularly during rotational and sagittal‐plane motion, the benefits of using two suture buttons remain uncertain [42]. Some studies have found no superior stability with a two‐button construct, whereas others have reported enhanced biomechanical stability in the sagittal and axial planes compared with a single‐button construct [17, 29, 31, 38, 46].

If a concomitant deltoid ligament rupture was identified during diagnostic arthroscopy, the medial gutter was debrided to expose the medial malleolus. A DX FiberTack Knotless Anchor (Arthrex) was then inserted into the anterior colliculus through an accessory medial portal. The blue suture was passed through the anterior and posterior portions of the deltoid ligament, after which the ligament was tensioned using the knotless system, as described by Cochonat and Lopes [11]. Syndesmotic stability was tested intraoperatively under fluoroscopy by using both the hook test and the lateral rotation test [55].

Postoperatively, the operated limb was immobilized in a walking boot for 1 month. Free passive and active mobilization were permitted immediately after surgery, while weight bearing was prohibited for 1 month postoperatively. All patients followed the standardized rehabilitation protocol presented in Table 1 for the first 6 postoperative weeks.

**Table 1:** Standardized postoperative rehabilitation protocol.

Postoperative period	Rehabilitation measures
0–2 weeks	‐Rest, ice, elevation, compression ‐Passive and active ROM exercises in the sagittal plane ‐Reinforcement exercises for quadriceps and hamstrings with the immobilization maintained
3–4 weeks	‐Passive and active ROM exercises in the sagittal plane with emphasis on dorsiflexion ‐Isometric reinforcement of the foot and calf muscles against resistance ‐Hydrotherapy and AlterG® Anti‐Gravity Treadmill™ (Lifeward) when possible
5–6 weeks	‐Progressive removal of immobilization ‐Progressive weight‐bearing, proprioception/balance exercises and gait training ‐Pursuit of resistance exercises in weight‐bearing positions ‐Active and passive mobilization in all planes taking care to avoid combined dorsiflexion and eversion ‐Hydrotherapy, stationary bike, AlterG® Anti‐Gravity Treadmill™ (Lifeward) with progressive resistance

Abbreviation: ROM, range of motion.

Postoperative follow‐up was conducted by the operating surgeon at 6 weeks and at 3 months. The 6‐week consultation included anteroposterior (AP) and lateral weight‐bearing ankle radiographs to assess maintenance of the reduction and verify the suture‐buttons fixation. Irrespective of the sports level (amateur, competitive, professional), the rehabilitation protocol applied by the physiotherapists generally followed the Pain, Ankle impairments, Athlete perception, Sensorimotor control and Sport/functional performance (PAASS) framework [53]. For both competitive and professional athletes, the team sports physician and/or the physiotherapist introduced specific exercises once the patient's perception and sensorimotor control allowed. These exercises included sport‐specific drills, plyometrics and high‐intensity functional exercises, with an emphasis on change‐of‐direction and deceleration drills [58, 60]. Depending upon the evolution and also the patient's will and readiness, the decision regarding RTS was multidisciplinary and made once the PAASS parameters were considered to be at a satisfactory level.

### The Ankle‐GO™ score

At 3 and 6 months, patients were evaluated using the Ankle‐GO^TM^ score and asked about RTS status. The response options were (a) no; (b) yes, but at a lower level of play or (c) yes, at a level equal to or higher than the pre‐injury level. Based on patient‐reported status, RTS at any level was defined as a return to unrestricted training and/or competition, including at a lower level, whereas RTS at the pre‐injury level was defined as a return to a level equal to or higher than the pre‐injury level. Data from the evaluations were collected through the Ankle‐GO^TM^ website (https://anklego.com). The score (Table 2) is reliable and has been validated as an outcome measurement tool according to the Consensus‐based Standards for the selection of health Measurement Instruments (COSMIN) for predicting RTS after lateral ankle sprains, Achilles tendon repairs and lateral ligament ankle reconstruction [23, 32, 46]. Its values range from 0 to 25 points.

**Table 2:** The Ankle‐GO™ score.

Test value	Weight	Maximum score by test
*Functional tests*		
SLS (errors)		
>3 errors	0	3
1–3 errors	1	
0 errors	2	
No feeling of instability	+1	
mSEBT, %		
<90	0	7
90–95	2	
>95	4	
Anterior > 60	+1	
Posteromedial > 90	+1	
No feeling of instability	+1	
SHT, s		
>13	0	5
10–13	2	
<10	4	
No feeling of instability	+1	
F8T, s		
>18	0	3
13–18	1	
<13	2	
No feeling of instability	+1	
*PROs*		
FAAM		4
FAAM‐ADL		
<90	0	2
90–95	1	
>95	2	
FAAM‐S		
<80	0	2
80–95	1	
>95	2	
ALR‐RSI		
<55	0	3
55–63	1	
63–76	2	
>76	3	

*Note*: Functional tests: SLS, mSEBT, SHT and F8T. Self‐reported questionnaires: FAAM with its two subscales ADL and S, ALR‐RSI.

Abbreviations: ADL, activity of daily living; ALR‐RSI, Ankle Ligament Reconstruction‐Return to Sport after Injury; FAAM, Foot and Ankle Ability Measure; F8T, figure‐of‐8 hop test; mSEBT, modified version of the Star Excursion Balance test in percentages; PROs, patient‐reported outcomes; S, sports activities; SHT, side hop test; SLS, single‐leg stance test.

**Figure 1:**
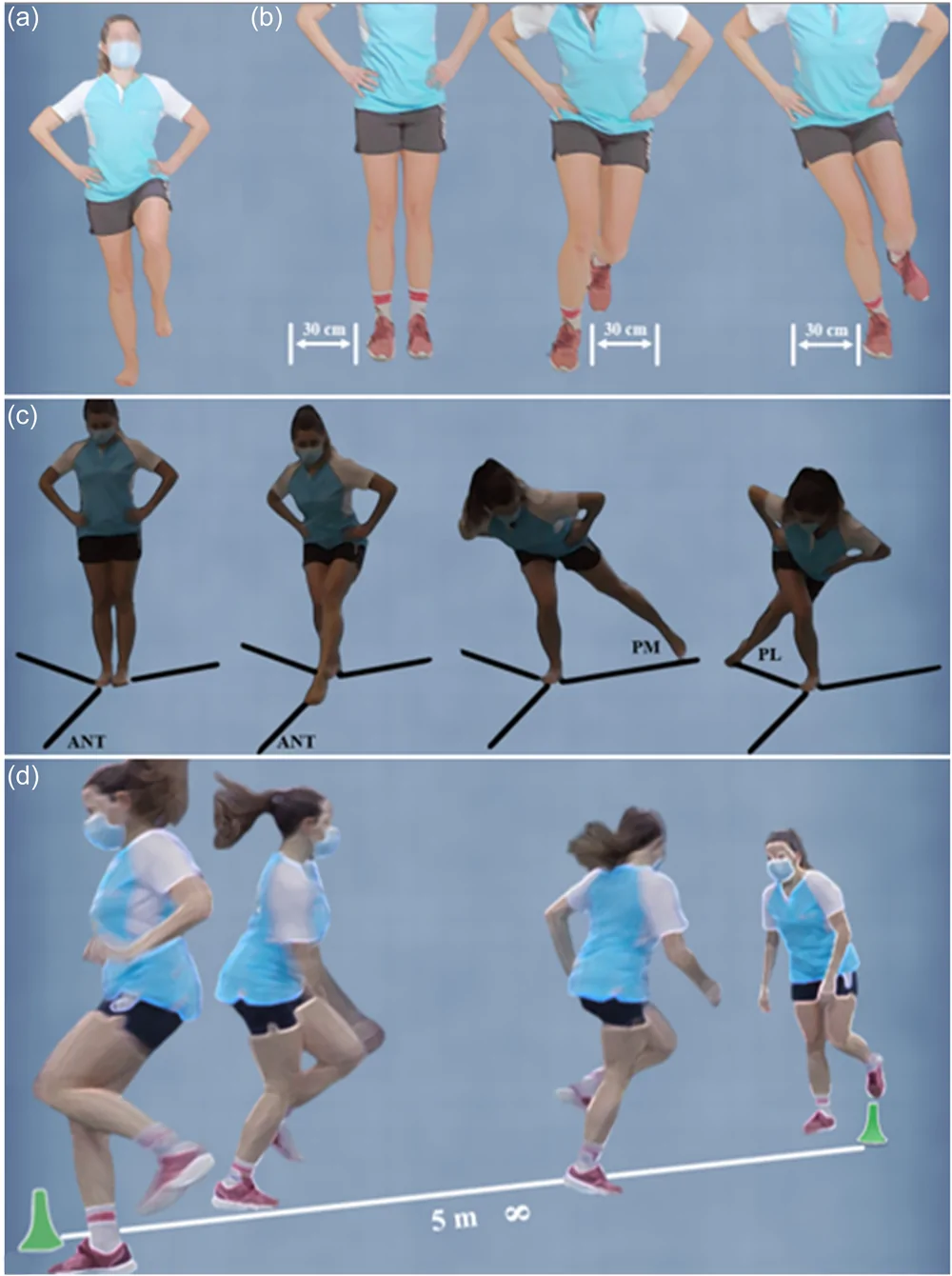
The four functional performance tests of the Ankle‐GO^TM^ score. (a) The single‐leg stance test on a firm surface (SLS Test). (b) The side hop test (SHT). (c) The modified version of the Star Excursion Balance Test (mSEBT); anterior direction (ANT); posteromedial direction (PM) and postero‐lateral direction (PL). (d) The figure‐of‐8 test (F8T).

As illustrated in Figure 1, the Ankle‐GO^TM^ score is composed of four functional performance tests: the Single‐Leg Stance on a firm surface (SLS test), the side hop test (SHT), the modified version of the Star Excursion Balance Test (mSEBT) and the figure‐of‐8 hop test (F8T). The score also includes two self‐reported questionnaires (Table 2). First, the Foot and Ankle Ability Measure (FAAM) is an instrument comprising two subscales that evaluate activities of daily living (FAAM‐ADL) and sports activities (FAAM‐S). It has been validated in patients with foot and ankle pathology, including athletic populations with chronic ankle instability [9, 35]. Second, since psychological factors play an important role in successful RTS after injuries, the Ankle Ligament Reconstruction‐Return to Sport After Injury (ALR‐RSI) questionnaire was added to the Ankle‐GO^TM^ score to offer a more complete evaluation of the patients' readiness for RTS [1, 5, 45, 48]. The ALR‐RSI was proven to be a valid, reproducible outcome measure tool to evaluate psychological readiness in patients who RTS after ankle fracture or lateral ligament reconstruction surgery, and it has been shown to be a stronger predictor of RTS in patients undergoing surgery for chronic ankle instability in comparison to American Orthopedic Foot & Ankle Society (AOFAS) and Karlsson scores [3, 20, 44, 48, 50, 51].

Each of the four functional tests assesses a specific component of balance, agility or dynamic postural control, as outlined below:

‐ The SLS test assesses static postural control depending on the number of errors committed by the subject. The standing position is assessed barefoot on a firm surface for 20 s, with the eyes closed, hands on the hips and knees slightly flexed. During this period, the evaluator counts the number of errors (lifting hands off hips, moving the thigh into more than 30° of flexion or abduction, lifting the forefoot or heel, remaining out of the testing position for more than 5 s or opening eyes). Two learning sessions should be allowed before assessing each foot.
‐ The mSEBT represents a simplified version of the Star Excursion Balance test [22, 47]. It assesses dynamic postural control by having the patient maintain a base of support on one leg while trying to reach the maximal distance in three directions (anterior, posteromedial and posterolateral) with the opposite leg while the hands are maintained on the hips. As part of the Ankle‐GO^TM^, the mSEBT is conducted in accordance with the updated recommendations [18]. The test per se begins after the examiner demonstrates the procedure and the subject performs four trials in each direction. After familiarization, the testing protocol involves measuring the distance from the standing centre to the spot on the tape that is touched by the opposite foot in each of the three directions. Three measurements are performed, and the mean value is calculated for each direction and each limb. Nonnormalized scores are expressed in centimeters, while normalized scores are expressed as percentages (proportion of mean values in each direction relative to the length of the lower limb measured from the anterior superior iliac spine to the medial malleolus).
‐ The SHT, which assesses neuromuscular function, requires the participant to perform 10 rapid lateral and medial hops using the injured limb above two lines placed 30 cm apart, without touching the lines [18].
‐ The F8T requires the participant to perform two laps in a figure‐of‐eight pattern between two cones separated by 5 m as fast as possible while hopping on one leg. Thus, the total completed distance is 20 m [7].

### Rationale for using the Ankle‐GO^TM^ score in syndesmotic injuries

**Figure 2:**
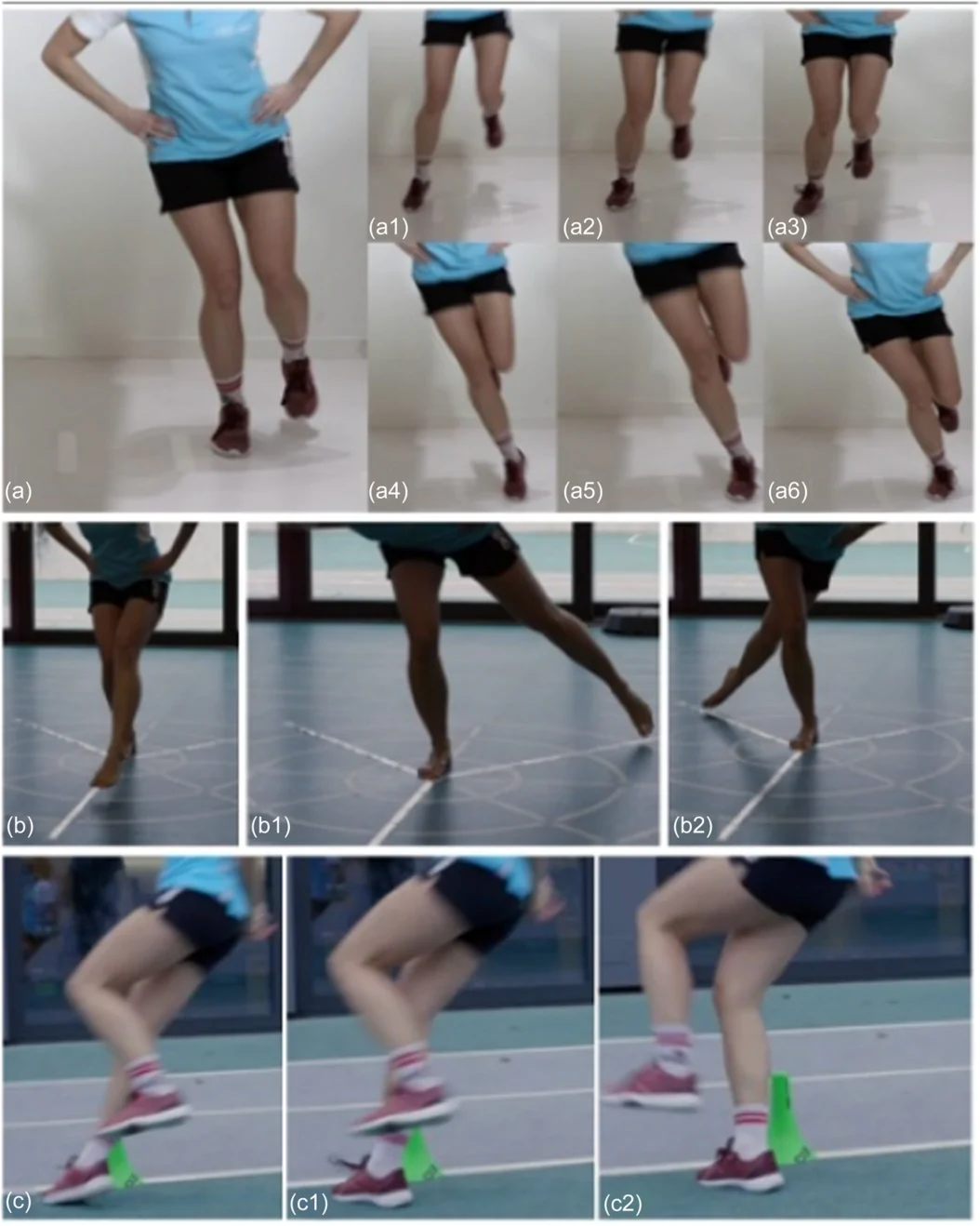
(a) Side hop test (SHT) starting position. (a1)–(a3) During lateral‐side landing, the ankle transitions from plantar flexion at initial contact to dorsiflexion, while the tibia internally rotates relative to the foot. (a4)–(a6) Similarly, during medial‐side landing, the ankle progresses from plantarflexion to dorsiflexion as the tibia rotates internally relative to the foot; the foot may also evert. (b) Modified Star Excursion Balance Test (mSEBT), anterior reach: The stance ankle is dorsiflexed as the participant reaches anteriorly. (b1) mSEBT, postero‐medial reach: The ankle of the support limb remains dorsiflexed while bearing axial load as the participant reaches postero‐medially. (b2) mSEBT, postero‐lateral reach: The stance ankle is dorsiflexed with slight foot eversion as the participant reaches postero‐laterally. (c) Figure‐of‐8 hop test (F8T): At initial ground contact, the ankle is axially loaded in plantar flexion, progresses through a neutral position (c1) and subsequently moves into dorsiflexion with foot eversion during stance (c2).

Although several mechanisms of syndesmotic injury have been described, the most important component is external rotation, which can be associated with dorsiflexion, plantar flexion, supination or pronation [40]. More rarely, dorsiflexion alone can induce a high ankle sprain by forcing the wider anterior part of the talar dome to act as a wedge against the mortise [10, 15]. In addition, an eversion mechanism can stress the syndesmosis, but only after the medial restraints have failed [10, 40]. The most commonly used clinical tests aim to reproduce pain by tensioning the syndesmotic ligaments and involve applying an external rotation force, a combination of external rotation and dorsiflexion, dorsiflexion alone or proximal calf compression, which increases the distal tibiofibular distance [39]. However, in addition to having limited diagnostic accuracy and interobserver reliability, these tests do not comprehensively evaluate proprioception, dynamic postural control, residual instability or psychological readiness for RTS [12, 57]. With the exception of the SLS test, the rest of the functional tests included in the Ankle‐GO^TM^ score place the ankle under weight‐bearing conditions that involve varying degrees of dorsiflexion, rotation, deceleration and multiplanar loading, thereby stressing the distal tibiofibular syndesmosis in a manner similar to that of the provocative clinical tests (Figure 2). During landing, foot eversion may also occur, thereby tensioning the deltoid ligament, whose lesion can be associated with syndesmotic pathology. Moreover, Mait et al. induced syndesmotic injuries with the ankle in both a neutral position and dorsiflexion while internally rotating the tibia to reproduce external rotation of the foot; these movement patterns can also occasionally be observed during the landing phase of the dynamic tests [34]. In addition to evaluating static and dynamic postural control, this score assesses the feeling of instability and psychological readiness, offering a more complex and functionally relevant perspective in comparison with clinical examination alone.

### Statistical analysis

Quantitative variables were summarized as means and standard deviations (SDs) when normally distributed and as medians with interquartile ranges (IQRs; 25th–75th percentiles) when nonnormally distributed. Categorical variables were summarized as frequencies and percentages. Normality of continuous variables was assessed using the Shapiro–Wilk test. As Ankle‐GO™ scores were not normally distributed, score responsiveness over time between 3 and 6 months was assessed using the Wilcoxon signed‐rank test for paired samples.

### Discriminative analysis

At 6 months, the ability of the Ankle‐GO™ score to discriminate between patients who returned to sport at any level and those who did not, as well as between patients who returned to sport at the pre‐injury level and those who did not, was evaluated using the Wilcoxon rank‐sum test.

### Predictive analysis

The predictive ability of the Ankle‐GO™ score at 3 months for RTS at 6 months was evaluated using both receiver operating characteristic (ROC) curve analysis and logistic regression. ROC curve analysis was used to calculate the area under the curve (AUC), the optimal cut‐off and predictive performance metrics (sensitivity, specificity, positive predictive value [PPV] and negative predictive value [NPV]). The optimal cut‐off was identified using the Youden index and was internally validated using bootstrap resampling (2000 iterations). This analysis was considered exploratory and aimed to assess the stability of the derived threshold. Logistic regression estimated odds ratios (ORs) with 95% confidence intervals (CIs) by comparing patients with scores above and below prespecified score thresholds, thereby quantifying the likelihood of returning to sport or returning to pre‐injury performance levels. Given the low number of non‐return events (*n* = 10) and evidence of quasi‐complete separation, Firth's penalized logistic regression was applied to reduce small‐sample bias and provide more stable effect estimates.

All statistical analyses were conducted using R software (Version 4.2, R Foundation for Statistical Computing).

## RESULTS

### Patient characteristics

A total of 50 patients with unilateral ligamentous syndesmotic lesions were included in the study. After 6 months, no patients were lost to follow‐up. Age, sex, body mass index (BMI), operated side, type of sport and level of sports participation are presented in Table 3.

**Table 3:** Characteristics of the study population.

	*N* (%)
Age at surgery, mean (SD)	30.2 (11.2)
Sex	
Female	12 (24.0%)
Male	38 (76.0%)
BMI, mean (SD)	25.6 (4.0)
Side	
Right	25 (50.0%)
Left	25 (50.0%)
Type of sport	
Football	18 (36.0%)
Rugby	11 (22.0%)
Running	5 (10.0%)
Dance	2 (4.0%)
Marching	2 (4.0%)
Judo	3 (6.0%)
Basketball	1 (2.0%)
Thai boxing	1 (2.0%)
Karate	1 (2.0%)
Fitness	1 (2.0%)
Gymnastics	1 (2.0%)
Badminton	1 ((2.0%)
Handball	1 (2.0%)
Triathlon	1 (2.0%)
Wakeboarding	1 (2.0%)
Level of sport	
Professional	25 (50.0%)
Competitive	11 (22.0%)
Recreational	14 (28.0%)

Abbreviations: BMI, body mass index; SD, standard deviation.

### General results of Ankle‐GO™ score at 3 and 6 months

The total score values and those of its individual components are presented in Table 4. The median Ankle‐GO™ score increased significantly from 13 points (IQR, 10.0–16.0) at 3 months to 19 points (IQR, 16.0–22.0) at 6 months (*p* < 0.0001). When analysing the individual components, all self‐reported questionnaires (FAAM‐ADL, FAAM‐S, ALR‐RSI) also showed significant improvement (*p* < 0.0001). Except for the SLS test, the remaining functional tests (mSEBT, SHT and F8T) demonstrated significant improvement from 3 to 6 months (*p* < 0.005).

**Table 4:** Results and comparison of the Ankle‐GO™ scores at 3 and 6 months.

	At 3 months	At 6 months	*p* value
Ankle‐GO™ score	13.0 (10.0–16.0)	19.0 (16.0–22.0)	<0.0001
FAAM‐ADL %	92.9 (88.1–96.4)	99.5 (94.0–100.0)	<0.0001
FAAM‐S %	75.0 (56.3–87.5)	96.9 (87.5–100.0)	<0.0001
ALR‐RSI %	61.3 (51.0–78.0)	84.6 (71.7–93.0)	<0.0001
SLS (errors)	1.0 (0.0–2.0)	1.0 (0.0–1.0)	0.9644
mSEBT %	88.1 (80.0–93.0)	94.3 (86.5–100.0)	0.0004
% ANT	65.9 (60.0–73.0)	69.7 (65.0–76.3)	0.0049
% PM	99.4 (88.0–107.5)	107.8 (97.8–113.4)	0.0015
% PL	98.0 (87.8–103.4)	104.8 (95.3–112.0)	0.0041
F8T, s	12.4 (11.0–15.0)	11.0 (10.0–13.3)	0.0006
SHT, s	12.0 (9.0–16.5)	10.0 (8.9–12.5)	0.0008

*Note*: Data are presented as median (interquartile range).

Abbreviations: ADL, activity of daily living; ALR‐RSI, Ankle Ligament Reconstruction‐Return to Sport after Injury; ANT, anterior direction; FAAM, Foot and Ankle Ability Measure; F8T, figure‐of‐8 hop test; mSEBT, modified version of the Star Excursion Balance test in percentages; PL, postero‐lateral direction; PM, posteromedial direction; S, sports activities; SHT, side hop test; SLS, single‐leg stance test.

As an additional analysis, the subcomponents of the Ankle‐GO™ score at 6 months were compared between patients who returned to sport and those who did not, both at any level (Table 5) and at the pre‐injury level (Table 6). Those participants who RTS at any level demonstrated statistically significant higher values of FAAM (both subscales) and ALR‐RSI questionnaires, as well as a faster completion of F8T and SHT.

**Table 5:** Comparison at 6 months of Ankle‐GO^TM^ score's subcomponents according to RTS at any level status.

	Return to sport at any level		
	No, *N* = 10	Yes, *N* = 40	*p* value
FAAM‐ADL %	94.0 (89.3–96.0)	100.0 (98.0–100.0)	<0.001
FAAM‐S %	75.0 (50.0–81.3)	100.0 (93.8–100.0)	<0.001
ALR‐RSI %	62.1 (39.2–75.0)	86.4 (73.6–94.6)	<0.001
SLS (errors)	0.0 (0.0–2.0)	0.0 (0.0–1.0)	0.6
mSEBT %	92.4 (82.3–95.6)	95.2 (89.9–101.5)	0.2
% ANT	62.6 (57.3–71.1)	70.5 (66.0–76.4)	0.075
% PM	106.8 (92.9–110.2)	108.1 (97.8–114.0)	0.5
% PL	100.6 (91.8–108.9)	106.0 (96.6–113.1)	0.4
F8T, s	14.5 (12.0–20.0)	10.9 (9.9–12.5)	<0.001
SHT, s	13.8 (10.0–25.0)	9.7 (8.2–11.4)	0.005

Abbreviations: ADL, activity of daily living; ALR‐RSI, Ankle Ligament Reconstruction‐Return to Sport after Injury; ANT, anterior direction; FAAM, Foot and Ankle Ability Measure; F8T, figure‐of‐8 hop test; mSEBT, modified version of the Star Excursion Balance test in percentages; PL, postero‐lateral direction; PM, posteromedial direction; S, sports activities; SHT, side hop test; SLS, single‐leg stance test.

**Table 6:** Comparison at 6 months of Ankle‐GO^TM^ score's subcomponents according to RTS at pre‐injury level status.

Return at pre‐injury level			
	No, *N* = 21	Yes, *N* = 29	*p* value
FAAM‐ADL %	94.0 (92.9–98.8)	100.0 (98.8–100.0)	<0.001
FAAM‐S %	81.3 (75.0–93.8)	100.0 (96.9–100.0)	<0.001
ALR‐RSI %	74.2 (57.5–88.0)	87.0 (77.0–95.8)	0.002
SLS (errors)	0.0 (0.0–1.0)	0.0 (0.0–1.0)	>0.9
mSEBT %	91.2 (85.7–94.7)	96.9 (92.9–104.5)	0.009
% ANT	68.0 (62.2–73.6)	71.0 (66.0–77.3)	0.14
% PM	101.1 (95.0–107.5)	111.1 (101.2–117.0)	0.01
% PL	100.0 (93.3–106.7)	108.2 (98.9–116.0)	0.07
F8T, s	12.0 (11.0–17.0)	10.9 (9.5–12.3)	0.007
SHT, s	11.0 (9.3–15.0)	9.8 (8.0–11.5)	0.058

Abbreviations: ADL, activity of daily living; ALR‐RSI, Ankle Ligament Reconstruction‐Return to Sport after Injury; ANT, anterior direction; FAAM, Foot and Ankle Ability Measure; F8T, figure‐of‐8 hop test; mSEBT, modified version of the Star Excursion Balance test in percentages; PL, postero‐lateral direction; PM, posteromedial direction; RTS, return to sport; S, sports activities; SHT, side hop test; SLS, single‐leg stance test.

Participants who returned to sport at their pre‐injury level had significantly higher FAAM‐ADL, FAAM‐S, ALR‐RSI and overall mSEBT than those who did not. They also completed the F8T significantly faster. No significant between‐group differences were observed for the SLS or SHT.

With regard to associated lesions, none of the patients had lateral ankle instability. As specified in the methodology, only unstable ligamentous syndesmotic lesions were included in this study. There were 13 (26%) intraoperatively confirmed associated deltoid ligament injuries, all of which were repaired arthroscopically. Table 7 illustrates the rates of RTS at the pre‐injury level according to the preoperative and intraoperative characteristics of the lesions. In the studied population, none of the parameters analysed (injury severity, individual lesions and the presence and/or repair of deltoid ligament injury) were significantly associated with successful RTS at pre‐injury level.

**Table 7:** Comparison of syndesmotic and deltoid ligament injury characteristics between patients who did and did not RTS at their pre‐injury level.

Characteristic	Overall (*N* = 50)	No RTS at pre‐injury level (*N* = 21)	RTS at pre‐injury level (*N* = 29)	*p* value
Syndesmotic grade, *n* (%)				>0.90
Grade IIB	32 (64.0%)	13 (61.9%)	19 (65.5%)	
Grade III	18 (36.0%)	8 (38.1%)	10 (34.5%)	
AITFL lesion, *n* (%)				—
Yes	50 (100.0%)	21 (100.0%)	29 (100.0%)	
PITFL lesion, *n* (%)				>0.90
No	32 (64.0%)	13 (61.9%)	19 (65.5%)	
Yes	18 (36.0%)	8 (38.1%)	10 (34.5%)	
Interosseous ligament lesion, *n* (%)				—
Yes	50 (100.0%)	21 (100.0%)	29 (100.0%)	
Deltoid ligament lesion on MRI, *n* (%)				>0.90
No	41 (82.0%)	17 (81.0%)	24 (82.8%)	
Yes	9 (18.0%)	4 (19.0%)	5 (17.2%)	
Intraoperative deltoid ligament lesion, *n* (%)				0.80
No	37 (74.0%)	15 (71.4%)	22 (75.9%)	
Yes	13 ((26.0%)	6 (28.6%)	7 (24.1%)	
Deltoid ligament lesion repaired, *n* (%)				—
Yes	13 (100.0%)	6 (100.0%)	7 (100.0%)	

Abbreviations: AITFL, anterior inferior tibiofibular ligament; PITFL, posterior inferior tibiofibular ligament; RTS, return to sport.

### Discriminative ability of the Ankle‐GO™ score

At 6 months, a total of 40 patients (80%) returned to sport, and 29 patients (58%) returned to sport at the pre‐injury level. At 6 months, participants who returned to sport, either at any level or at the pre‐injury level, had significantly higher Ankle‐GO™ scores than the corresponding non‐returning groups (21.0 [IQR: 18.5–22.0] vs. 12.0 [IQR: 11.0–17.0]; *p* < 0.001 and 21.0 [IQR: 19.0–22.0] vs. 16.0 [IQR: 13.0–19.0]; *p* < 0.001), indicating good discriminative ability of the Ankle‐GO™ score (Table 8).

**Table 8:** Discriminative ability of the Ankle‐GO™ score at 6 months for RTS and RTS at pre‐injury level.

Outcome at 6 months	*N*	Ankle‐GO™ score	*p* value
Return at any level	No = 10 (20%)	12.0 (11.0–17.0)	<0.001
	Yes = 40 (80%)	21.0 (18.5–22.0)	
Return to pre‐injury level	No = 21 (42%)	16.0 (13.0–19.0)	<0.001
	Yes = 29 (58%)	21.0 (19.0–22.0)	

Abbreviation: RTS, return to sport.

### Predictive ability of the Ankle‐GO™ score

**Figure 3:**
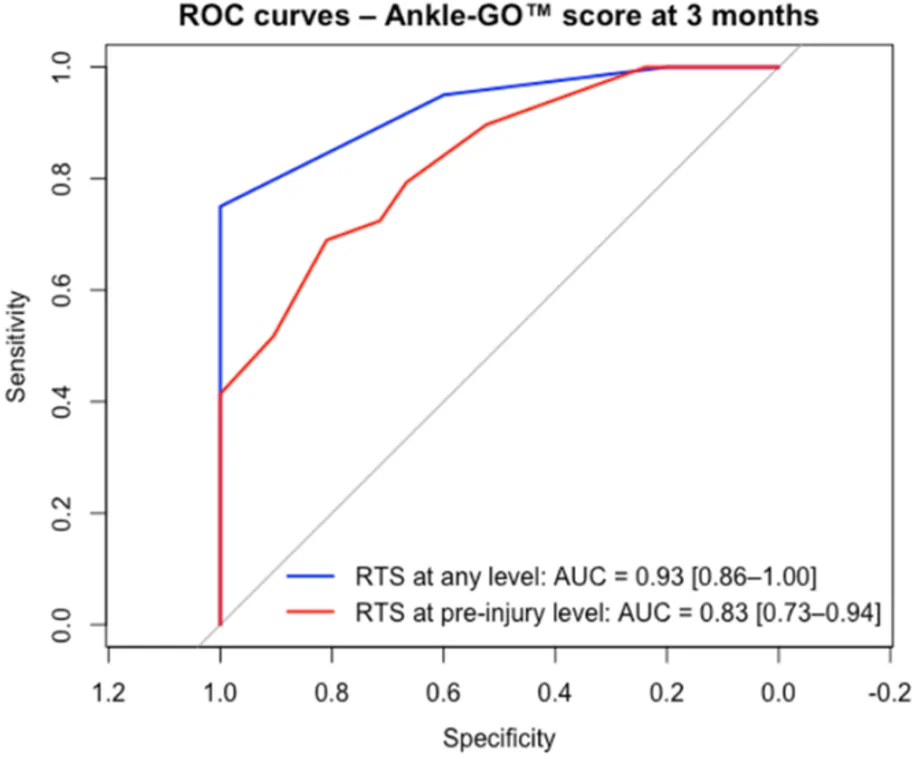
ROC curve of Ankle‐GO™ score at 3 months for RTS at 6 months. AUC, area under the curve; ROC, receiver operating characteristic curve; RTS, return to sport.

Regarding RTS at any level, the Ankle‐GO™ score at 3 months showed excellent predictive ability, with an AUC of 0.93 (95% CI: 0.86–1.00) and a PPV of 100%, indicating that all patients scoring above 12 points returned to sport at any level at 6 months (Table 9 and Figure 3). This threshold also yielded 75% sensitivity and 100% specificity. A cut‐off value of 14 points at 3 months was a good predictor of RTS at the pre‐injury level at 6 months, with an AUC of 0.83 (95% CI: 0.73–0.94). However, the score was less reliable in identifying patients who would not return, with NPVs of 50% for RTS at any level and 65% for RTS at the pre‐injury level. Internal validation using bootstrap resampling (2000 iterations) suggested good stability of the estimated thresholds, with median optimal cut‐offs based on the Youden index of 11.5 (95% CI: 8.0–12.0) for RTS at any level and 13.5 (95% CI: 10.0–16.0) for RTS at the pre‐injury level.

**Table 9:** Predictive performance of the Ankle‐GO™ score (3‐month score predicting 6‐month sport status).

	Predictive ability of the Ankle‐GO™ score at 3 months								
Return to sport at 6 months	Youden's threshold	TP	TN	FP	FN	Sensitivity	Specificity	PPV	NPV
At any level	12	30	10	0	10	75%	100%	100%	50%
At pre‐injury level	14	20	17	4	9	69%	81%	83%	65%

Abbreviations: FN, false negative; FP, false positive; NPV, negative predictive value; PPV, positive predictive value; TN, true negative; TP, true positive.

Patients with an Ankle‐GO™ score ≥12 at 3 months had increased odds of returning to any level of sport (OR = 42.78, 95% CI: 2.33–785.93, *p* < 0.01), while those with a score ≥14 had 9.44 times higher odds of returning at their pre‐injury level by 6 months compared to those with scores below 14 points (OR = 9.44, 95% CI: 2.66–40.7, *p* < 0.01) as illustrated in Table 10.

**Table 10:** Logistic regression assessing the association between Ankle‐GO^TM^ score at 3 months and RTS at 6 months.

RTS at 6 months	Ankle‐GO^TM^ threshold	Odds ratio	95% Confidence interval	*p* value
At all levels	≥12	42.78	2.33–785.93	<0.01
At pre‐injury level	≥14	9.44	2.66–40.7	<0.01

Abbreviation: RTS, return to sport.

## DISCUSSION

The results of this study provide preliminary evidence supporting the discriminative and predictive ability of the Ankle‐GO™ score for RTS in patients who underwent dynamic surgical stabilization for unstable syndesmotic ankle injuries.

The median Ankle‐GO™ score increased by 6 points from 3 to 6 months, which was greater than the minimal detectable change reported in the initial validation study of the score in patients with lateral ankle sprains, suggesting that patients surgically treated for syndesmotic injuries experienced a real clinical improvement [46]. Furthermore, the median value of 19 points at 6 months in the present study was higher than previously reported values in patients who underwent lateral ankle reconstruction (15.5 ± 4.5), patients who underwent Achilles tendon repair (10.7 ± 4.8), and patients with lateral ankle sprains who returned to sport after completing a 4‐month rehabilitation protocol (17.2 ± 3.5) [23, 32, 46]. This suggests that the postoperative programme in the present study may be sufficient to achieve satisfactory functional recovery by 6 months. In the current study, the SLS test was the only functional parameter that did not show a statistically significant improvement between 3 months (median, 1.0 errors; IQR, 0.0–2.0) and 6 months (median, 1.0 errors; IQR, 0.0–1.0; *p* = 0.964). Doherty et al. reported 2.9 ± 2.1 errors in patients with functional ankle instability and 1.6 ± 1.3 errors in controls, whereas Picot et al. reported 2 ± 4 errors in lateral ankle sprain copers at 2 months [19, 43]. This suggests that satisfactory static postural control may already have been achieved by 3 months following the recommended postoperative rehabilitation protocol, with little additional improvement occurring between 3 and 6 months in patients undergoing dynamic surgical stabilization for syndesmotic injuries.

The score exhibited excellent discriminative performance in distinguishing patients who returned to sport at any level from those who did not return. For this population, a cut‐off value of 12 points yielded a sensitivity of 75%, indicating that the score correctly identified three‐quarters of patients who returned to sport at 6 months, irrespective of participation level (recreational, competitive or professional). The score also correctly identified all patients who did not RTS, as reflected by a specificity of 100%. For RTS at the same or a higher level of play, the AUC values of 0.83 at both 3 and 6 months reflect good discriminative ability, though somewhat lower than for RTS at any level. The lower accuracy in identifying athletes who returned to sport at the pre‐injury level suggests that, beyond physical recovery, psychological factors may play an important role. The improvement in the ALR‐RSI scale to a median value of 84.6% (IQR: 71.7–93.0) at 6 months for this subset of patients (Table 4) suggests that psychological readiness may have contributed to the observed difference in discriminative ability between the two subgroups. Given that the ALR‐RSI has been validated as a reliable tool for assessing psychological readiness for RTS in active populations following ankle fractures, the use of ALR‐RSI to identify and support athletes with syndesmotic injuries who experience psychological barriers to RTS during the first 3 postoperative months could improve rates of RTS at the pre‐injury level [50, 51]. However, its measurement properties should also be confirmed specifically in patients with syndesmotic injuries.

Overall, the Ankle‐GO™ score appeared to perform better in predicting RTS at any level than in predicting RTS at the pre‐injury level, as evidenced by higher PPV and sensitivity values for the former outcome. Specifically, patients with Ankle‐GO™ scores ≥12 had 43 times the odds of RTS at any level, whereas scores ≥14 were associated with nine‐fold higher odds of returning to sport at the pre‐injury levels. At 3 months, the score showed a perfect PPV (100%), as all patients scoring ≥12 returned to sport at 6 months. Despite moderate NPVs (50%–65%), the high specificity values (100% for RTS at any level and 81% for RTS at the pre‐injury level) indicate that patients who did not RTS were rarely incorrectly classified as likely to return.

To our knowledge, this is the first study to assess the responsiveness and predictive and discriminative abilities of an outcome measurement instrument for RTS after dynamic fixation of unstable ligamentous syndesmotic injuries. Reported RTS rates, timing and patient‐reported outcome measures (PROMs) vary in the literature. D'Hooghe et al. reported a 47% RTS rate at 2 months and a 95% rate of return to match play at 6 months in professional football players operated for grade IIB and III unstable ligamentous syndesmotic injuries, whereas a meta‐analysis found a 100% RTS rate at 7 weeks in elite athletes who underwent surgery for this pathology [6, 16]. In the present study, 40 patients (80%) returned to sport, and 29 patients (58%) returned to their pre‐injury level at 6 months. The observed difference may partly reflect variations in the study populations, as only 50% of patients in the present study were professional athletes. Colcuc et al. reported a 100% RTS rate at 14 weeks after dynamic syndesmotic fixation, whereas Laflamme et al. observed more gradual RTS rates despite reporting similar AOFAS scores at 6 and 12 months [12, 30]. Although both studies included patients with concomitant ankle fractures and are not directly comparable with the present study, their findings illustrate the heterogeneity of RTS timing and rates despite broadly similar reported functional outcomes.

Nevertheless, several limitations should be acknowledged. First, the study population was relatively small for the validation of a prognostic score; however, cohorts of patients with syndesmotic injuries are generally limited in size because of the lower incidence of these lesions. As this was an exploratory study, no formal sample size calculation was performed; instead, the sample was determined by the number of eligible patients available during the study period. Accordingly, the large OR for RTS at any level should be interpreted with caution because of the wide CI. We also acknowledge that the limited sample size did not allow adjustment for relevant variables, such as pre‐injury level of sports participation, which may have influenced the observed associations. An additional limitation is the potential influence of concomitant deltoid ligament repair on postoperative recovery and functional outcomes. Although the number of patients who underwent deltoid ligament repair was reported, the limited sample size did not allow a separate subgroup analysis; therefore, the potential effect of this procedure on the results cannot be excluded. Although the Ankle‐GO^TM^ score demonstrated discriminative and predictive abilities in this cohort, external validation in independent populations is necessary. The two‐centre design and the inclusion of a heterogeneous sample with respect to sport type and competitive level enhance the external validity of the Ankle‐GO^TM^ score. However, the inclusion of professional, competitive and recreational athletes may have influenced outcomes and RTS rates because of potential differences in motivation, access to physiotherapy and performance demands. Although a longer follow‐up period might have affected the responsiveness and predictive ability of the Ankle‐GO^TM^ score, the study was designed to assess both of these properties at 6 months because this time point corresponds to the expected RTS period for most athletes. The predictive performance of the composite Ankle‐GO^TM^ score was not compared with that of its individual components; future studies should perform this comparison. The present study did not include standardized objective imaging assessments at the 3‐ and 6‐month follow‐up visits. Although intraoperative stress testing and routine radiographs obtained at 6 weeks indicated satisfactory syndesmotic reduction and showed no evidence of fixation failure, we could not assess subtle residual syndesmotic malreduction or examine the association between anatomical healing and the Ankle‐GO™ score. Taking these aspects into consideration, the score's predictive value may reflect functional adaptation, anatomical stability or both. Finally, rehabilitation after the initial 6‐week postoperative protocol was criterion‐based and individualized according to each patient's clinical recovery and sport‐specific demands. Although all patients followed the PAASS framework, variations in rehabilitation progression may have influenced the results of the 3‐month functional assessment.

## CONCLUSION

The Ankle‐GO™ score is the first predictive clinical outcome measurement instrument to evaluate surgically treated unstable ligamentous syndesmotic injuries; it demonstrated good responsiveness and excellent discriminative ability in identifying patients who returned to sport by 6 months after surgery. In addition, the score showed a good ability to predict RTS at the pre‐injury level. At 3 months, all of the patients with a score of at least 12 points returned to sport at any level by 6 months, whereas 83% of those with a minimum score of 14 points returned to their pre‐injury level within this timeframe. Taken together, these findings support the potential utility of this tool for predicting RTS after syndesmotic fixation. Further studies with larger sample sizes are needed to confirm these findings as well as the score's ability to predict the risk of reinjury.

## AUTHOR CONTRIBUTIONS

**Andrei Corbu**: Interpretation of results; manuscript writing and editing; literature review. **Ronny Lopes**: Conception and design of the study; patient inclusion and data collection; interpretation of results; critical review; manuscript writing. **Alexandre Hardy**: Conception and design of the study; manuscript writing; patient inclusion and data collection; statistical analysis; interpretation of results; critical review. **Eugénie Valentin**: Study design; data collection; statistical analysis; drafting of tables and figures; writing and interpretation of results. **Mette Renate Andersen**: Interpretation of results; critical review; manuscript writing. **Brice Picot**: Interpretation of results; manuscript writing; critical review. **François Fourchet**: Interpretation of results; literature review; manuscript writing. **Gauthier Rauline**: Data collection; literature review.

## FUNDING INFORMATION

The authors have no funding to report.

## CONFLICT OF INTEREST STATEMENT

Alexandre Hardy serves as a consultant for Arthrex and Groupe FH. Ronny Lopes serves as a consultant for Arthrex, contributes to development for ENOVIS and provides consulting and development services for STEPS ORTHO and EVOLUTIS. Mette Renate Andersen serves as a board member of ESSKA‐AFAS. The remaining authors declare no conflict of interest.

## ETHICS STATEMENT

This study received approval from Conseil d'Orientation Scientifique Ramsay Santé, Comité d'Ethique—IRB (approval reference COS‐RGDS‐2025‐07‐005‐HARDY‐A). Informed consent was obtained from the participants at the time of initial consultation.

## Data Availability

Data are available upon request.
